# MM-associated circular RNA downregulates microRNA-19a through methylation to suppress proliferation of pancreatic adenocarcinoma cells

**DOI:** 10.1080/21655979.2022.2051815

**Published:** 2022-04-07

**Authors:** Xinye Qian, Wenru Zong, Liqing Ma, Zhoujing Yang, Wei Chen, Jun Yan, Jianghui Xu

**Affiliations:** a Center of Hepatobiliary Pancreatic Disease, Beijing Tsinghua Changgung Hospital, School of Clinical Medicine, Tsinghua University, Beijing City, PR. China; b Department of Anesthesiology, Huashan Hospital, Fudan University, Shanghai City, PR. China; c Department of Anaesthesiology, Fudan University Shanghai Cancer Center, Department of Oncology, Shanghai Medical College, Fudan University, Shanghai City, PR. China

**Keywords:** Pancreatic adenocarcinoma, circ-MYBL2, miR-19a, methylation

## Abstract

Opposite roles of circular RNA MM-associated circular RNA (circ-MYBL2) have been observed in different malignancies, and its role in pancreatic adenocarcinoma (PA) is unknown. Our preliminary sequencing data revealed its inverse correlation with microRNA-19a (miR-19a). This study was performed to explore the role of circ-MYBL2 in PA and its crosstalk with miR-19a. The accumulation of circ-MYBL2 and miR-19a in PA was detected by RT-qPCR. Participation of circ-MYBL2 in the regulation of miR-19a and its RNA gene methylation was studied with an overexpression assay, followed by RT-qPCR and MSP analyses. The role of miR-19a and circ-MYBL2 in PA cell proliferation and movement was evaluated using the BrdU assay and the Transwell assay, respectively. Downregulation of circ-MYBL2 and upregulation of miR-19a were observed in PA. In PA cells, circ-MYBL2 decreased the accumulation of miR-19a but increased its RNA gene methylation. Overexpression of circ-MYBL2 decreased PA cell proliferation and movement, while overexpression of miR-19a showed an opposite effect. In addition, circ-MYBL2 suppressed the role of miR-19a in cell proliferation, migration, and invasion. In conclusion, circ-MYBL2 was downregulated in PA and it downregulated miR-19a through methylation to suppress PA cell proliferation.

## Graphical Abstract

**Figure:**
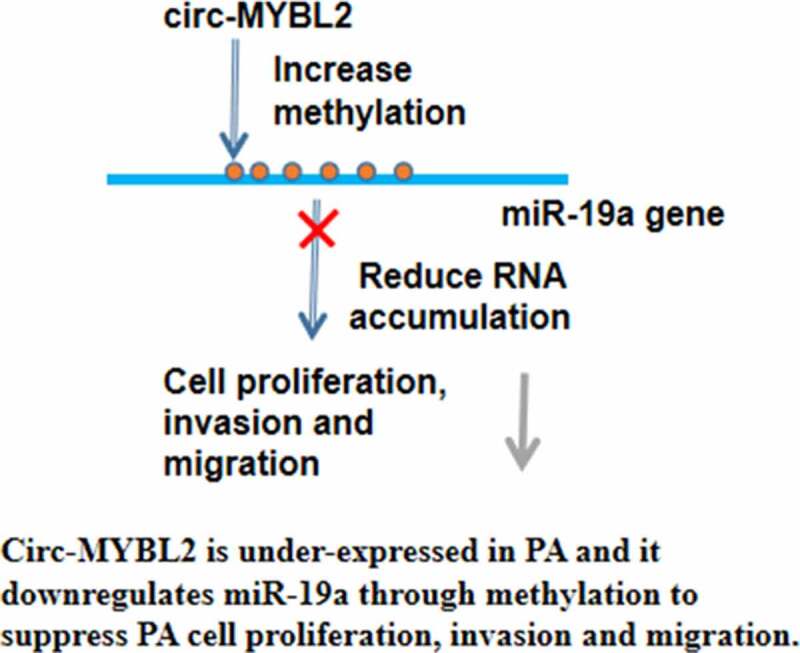

## Introduction

Pancreatic adenocarcinoma (PA), which also refers to ductal carcinoma and adenocarcinoma, accounts for more than 90% of pancreatic cancers [1]. Although PA is not a frequently diagnosed malignancy in clinical practice, it is a major cause of cancer-related deaths, mainly owing to high mortality rate [2,3]. It is estimated that PA affects 2.8–7.2 per l00,000 people across different populations [^2–4^]. However, PA is rarely curable, and only 5–10% of PA patients can achieve long-term (5-year) survival [5].

Treatment of PA requires novel therapies. With the advantages of high specificity and less side effects, targeted therapies, which aim to regulate gene expression to suppress tumor development, are emerging novel therapies for cancers, especially metastatic cancer treatment [^6–8^]. For instance, certain signaling pathways, such as TRK and CDK4/6, can be targeted to treat PA [9,10]. However, more studies are needed to screen for targets with higher efficiency and safety. Circular RNAs (circRNAs) are RNA transcripts with no or limited capacity of protein-coding, but regulate gene expression to mediate cancer [11]. Therefore, circRNAs are potential targets for the treatment of PA. MM-associated circular RNA MYB proto-oncogene like 2 (circ-MYBL2) has been shown to exert different roles in different cancers [12,13], while its role in PA is unknown. Our preliminary sequencing data revealed an inverse correlation between circ-MYBL2 and microRNA-19a (miR-19a), which is also a critical player in many cancers including PA [14]. We then speculated that circ-MYBL2 and miR-19a may interact with each other to regulate PA. This study explored the role of circ-MYBL2 in PA, with a focus on its interaction with miR-19a.

## Materials and methods

### Sample information

A total of 50 pairs of PA samples and matched tumor-adjacent normal tissues were provided by 59 PA patients (25 females and 34 males, 50.2 ± 5.6 years old, 28 cases of stage I/II, and 31 cases of stage III/IV) who were admitted at Huashan Hospital, Fudan University between May 2018 and May 2020. The Ethics Committee of this hospital approved this study. All patients signed the informed consent. Surgical resection was performed for all patients. All samples were confirmed by two independent pathologists through histopathological exams.

### Cell culture

Human PA cells SW1990 and PANC-1 (ATCC) were used in this study. The DMEM medium (Gibco, USA) supplemented with FBS (10%), penicillin (100 U/mL), and streptomycin (100 mg/mL) was used to cultivate the cells at 37°C with 5% CO_2_ in an incubator.

### Vectors and transfection

SW1990 and PANC-1 cells were overexpressed with circ-MYBL2 and/or miR-19a through the transfection of circ-MYBL2 (pcDNA3.1) expression vector and/or mimic of miR-19a (RiboBio). Transfection was performed with Neon Electroporation Transfection device (Thermo Fisher Scientific). In each transfection, 10^6^ cells were transfected with either 10 µg vector or 50 nM miRNA. After transfection, cells were subjected to cell culture in fresh medium for a total of 48 h prior to the subsequent assays.

### RNA isolation and RT-qPCR

Direct-Zol reagent (ZYMO Research) was used to isolate total RNAs. Digestion of genomic DNA was performed with DNase I (RiboBio). RNA integrity and concentration were determined using a BioAnalyzer. All samples had an RIN value higher than 8.5, indicating high RNA integrity. The preparation of cDNA samples was performed with 3 µg total RNAs as template. Expression of circ-MYBL2(Fr: TGTTAAGACCCTGCCC TTCTC; Re: CAGGACTTGCTGCTGATGTGAC) was determined through qPCR. Expression of miR-19a(Fr: GCGTGTGCAAATCTATGCA; Re: AGTGCAGGGTCCGAGGTATT) was also determined through RT-qPCR with U6 (Fr: CTCGCTTCGGCAGCACA; Re: AACGCTTCA CGAATTTGCGT) as an internal control. Ct values were normalized using the 2^−ΔΔCt^ method [15].

### BrdU assay

BrdU incorporation, which directly reflects DNA synthesis, was used to analyze cell proliferation. Briefly, the transfected cells were harvested at 48 h post-transfection and further incubated with 10 µM BrdU for 48 h. After that, cells were fixed for 30 min and incubated with peroxidase-coupled anti-BrdU-antibody (Sigma-Aldrich) for 60 min. After washing for three times with PBS, incubation with tetramethylbenzidine was performed for 30 min. Finally, the absorbance at 450 nm was measured to assess the ability of cells to proliferate. Negative control cells were treated with BrdU but not its antibody.

### Wound healing assay

For the scratch wound healing assay, SW1990 and PANC-1 cells were seeded into plastic six-well plates at the density of 5 × 10^5^ cells per well and cultured for 12 h. When a confluence of 80% was reached, the medium was discarded, and uniform scratch wounds were scraped by a sterile pipette tip. Each well was washed with PBS, and then supplemented with basal DMEM. Images of each scratch were observed under a microscope and captured at 0 and 24 h after scratching.

### Transwell assay

Upper chamber was used to cultivate cells in a non-serum medium. The lower chamber containing 10% FBS was used to serve as chemoattractant. After 48 h of incubation, the migrated or invaded cells were fixed with 90% methanol and stained with 0.1% crystal violet (Sigma-Aldrich). Coated and uncoated membranes were used for migration and invasion assays, respectively.

### DNA methylation assay

Cells with transfection were used to isolate genomic DNA through conventional methods. DNA samples were converted using sodium bisulfate (EZ DNA Methylation-Gold TM Kit, Qiagen). Then, the methylation of miR-19a promoter region was analyzed by methylation-specific PCR (MSP) method.

### Western blot analysis

Total proteins from different groups were extracted with Trizol with protease inhibitors. Protein concentrations were determined using a BCA kit (Abcam, USA). Next, protein samples were separated by SDS-PAGE, and transferred onto PVDF membranes (BioRad, Hercules, CA). The membranes were incubated with the primary antibodies against GAPDH (1:1,000, Abcam), MMP2 (1:1,500, Abcam), MMP9 (1:1,000), and VEGF (1:1,000, Abcam) at 4°C for 12 h. After blocking in 5% nonfat milk for 1 h, the membranes were incubated with secondary antibodies at room temperature for 1 h and washed with Tris-buffered saline with Tween 20 (TBST). The signals were visualized using an enhanced chemiluminescence (ECL) reagent.

### Statistical analysis

All experiments were performed with three biological replicates. Data were presented as the mean ± standard deviation (SD). Paired tissues were compared using paired t-test. Multiple independent groups were compared using ANOVA Tukey’s test. Correlation analysis was carried out with Pearson’s correlation coefficient. *P* < 0.05 was statistically significant.

## Results

### Expression of circ-MYBL2 and miR-19a in PA

**Figure 1.**
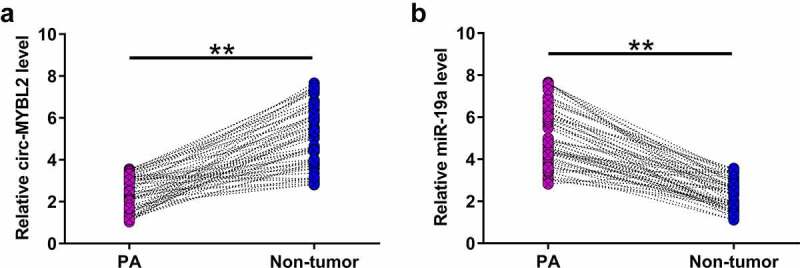
The expression of circ-MYBL2 and miR-19a in PA. Expression analysis of circ-MYBL2 (a) and miR-19a (b) in paired PA and non-tumor tissues from 59 PA patients was performed with RT-qPCR. qPCR was repeated for three times and average values are presented here for comparisons.

Altered expression may indicate gene function. Therefore, the expression of circ-MYBL2 and miR-19a in samples from 59 PA patients was performed with RT-qPCR. The results showed that circ-MYBL2 was downregulated in PA tissues in most samples from PA patients (Figure 1a, *p* < 0.01). MiR-19a was highly upregulated in PA samples (Figure 1b, *p*< 0.01). These results suggested the involvement of circ-MYBL2 and miR-19a in PA.

### The correlation between circ-MYBL2 and miR-19a

**Figure 2.**
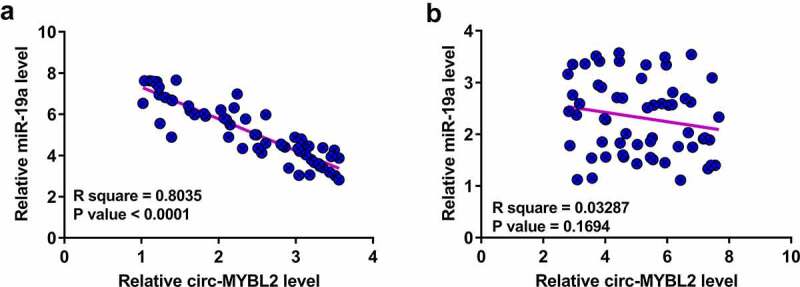
Correlation between circ-MYBL2 and miR-19a across PA tissues and non-tumor tissues. Correlations between circ-MYBL2 and miR-19a across PA tissues (a) and non-tumor (b) tissues were subjected to Pearson’s correlation coefficient analysis.

Our preliminary sequencing analysis revealed a close correlation between circ-MYBL2 and miR-19a. Pearson’s correlation coefficient analysis showed that circ-MYBL2 and miR-19a were inversely correlated across PA tissues (Figure 2a). However, they were not correlated across non-tumor tissues (Figure 2b). Therefore, circ-MYBL2 and miR-19a may interact with each other in PA.

### Regulatory role of circ-MYBL2 in miR-19a expression and its promoter region methylation

**Figure 3.**
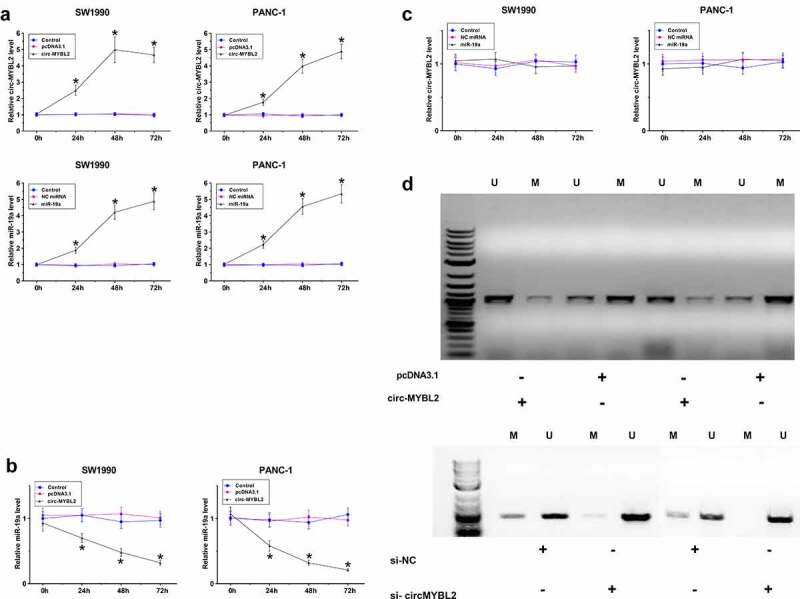
The role of circ-MYBL2 in the expression of miR-19a and its promoter region methylation. SW1990 and PANC-1 cells were overexpressed with circ-MYBL2 or miR-19a, followed by the confirmation of overexpression of circ-MYBL2 and miR-19a by RT-qPCR every 24 h until 96 h (a). Regulatory role of circ-MYBL2 in miR-19a expression (b) and regulatory role of miR-19a in circ-MYBL2 expression (c) were explored by RT-qPCR. The role of overexpression and silencing of circ-MYBL2 in promoter region methylation of miR19a was analyzed with MSP (d). *, *p* < 0.05.

To further confirm the interaction between circ-MYBL2 and miR-19a in PA, SW1990 and PANC-1 cells were overexpressed with circ-MYBL2 or miR-19a, followed by the confirmation of overexpression using RT-qPCR every 24 h until 96 h (Figure 3a, *p* < 0.05). It showed that overexpression of circ-MYBL2 decreased the expression levels of miR-19a (Figure 3b, *p* < 0.05). However, overexpression of miR-19a did not alter the expression of circ-MYBL2 (Figure 3c, *p* < 0.05). The role of circ-MYBL2 in regulating promoter region methylation of miR19a was assessed by MSP. Compared to transfection with empty pcDNA3.1 vector, transfection with the circ-MYBL2 vector increased methylation of miR-19a RNA gene (Figure 3d). We therefore concluded that circ-MYBL2 may increase miR-19a RNA gene methylation to downregulate miR-19a RNA.

### The role of circ-MYBL2 and miR-19a in the proliferation and movement of SW1990 and PANC-1 cells

**Figure 4.**
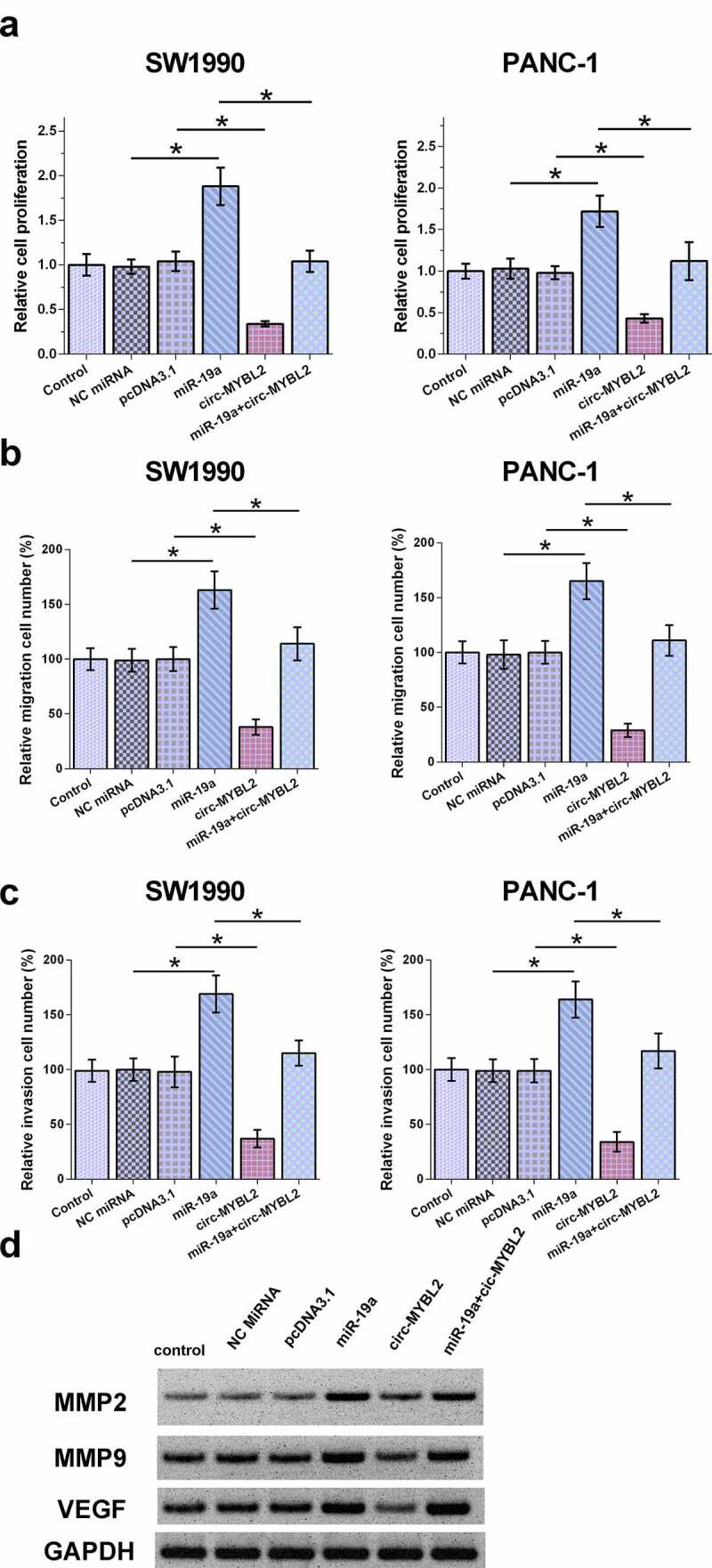
Analysis of the role of circ-MYBL2 and miR-19a in the proliferation of SW1990 and PANC-1 cells. (a) BrdU assays were performed to analyze the role of circ-MYBL2 and miR-19a in the proliferation of SW1990 and PANC-1 cells. Cell migration (b) and invasion (c) was detected through Transwell assay. The Western blot result of VEGF, MMP2, and MMP9 (d). *, *p* < 0.05.

Cell behavior determines cancer progression. Therefore, BrdU and Transwell assays were performed to evaluate the role of circ-MYBL2 and miR-19a in regulating the behavior of PA cells. BrdU analysis showed that circ-MYBL2 decreased proliferation, while miR-19a increased proliferation of PA cells. In addition, circ-MYBL2 suppressed the role of miR-19a in cell proliferation (Figure 4a). Circ-MYBL2 decreased cell movement, but the transfection of miR-19a led to an apparent promotion of cell movement. Moreover, our results demonstrated that circ-MYBL2 antagonized the role of miR-19a on the movement of SW1990 and PANC-1 cells (Figure 4b–C). Similar results were also observed in wound healing assay (Supplement Figure 1). To verify the effect of overexpression of miR-19a on the behavior of PA cells, the expression of three related characteristic proteins MMP2, MMP9, and VEGF was also detected. The effect of overexpression of miR-19a also increased the expression levels of MMP2, MMP9, and VEGF, while circ-MYBL2 can partially rescue the effect (Figure 4d). Therefore, circ-MYBL2 may suppress the role of miR-19a in inhibiting cancer cell proliferation and metastasis.

## Discussion

This study investigated the role of circ-MYBL2 in PA and its potential crosstalk with miR-19a. We observed that circ-MYBL2 was downregulated in PA and played tumor suppressive roles by inhibiting cancer cell proliferation. Moreover, the role of circ-MYBL2 in PA was likely achieved by downregulating miR-19a through methylation.

To date, the functionality of circ-MYBL2 has only been investigated in two types of cancer including cervical cancer and multiple myeloma, while the roles of circ-MYBL2 in these two types of cancer are opposite. Cervical cancer tissues exhibited increased expression levels of circ-MYBL2 and overexpression of circ-MYBL2 increased cell invasion and proliferation by sponging miR-361-3p [12]. In contrast, downregulation of circ-MYBL2 was observed in multiple myeloma [13]. Overexpression of circ-MYBL2 in multiple myeloma cells downregulates the expression of proliferation-related oncogenes by increasing the binding of Cyclin F to MYBL2, which inhibits the activation and phosphorylation of MYBL2. The present study revealed the downregulation of circ-MYBL2 in PA.

MiR-19a plays tumor suppressive roles in different types of cancer, including PA [14]. In PA, miR-19a is upregulated and downregulates Ras homolog family member B (RHOB) to suppress cancer cell apoptosis [14]. This study confirmed the upregulation of miR-19a in PA. In addition, we also showed that miR-19a may also play oncogenic roles in PA by increasing cell proliferation, migration, and invasion.

However, the upstream regulator of miR-19a in cancer biology is barely known. In the present study, we showed that circ-MYBL2 could suppress the expression of miR-19a by increasing the methylation level of its promoter region. Therefore, circ-MYBL2 may downregulate miR-19a through methylation pathways to play tumor suppressive roles, while the methylation factors involved in this process remain to be identified.

With the increased elucidation of the molecular mechanisms involved in PA, an increasing number of factors with critical function have been identified in PA [16,17]. These factors are potential targets for treating PA.

## Conclusion

Circ-MYBL2 is downregulated in PA and plays tumor suppressive roles by inhibiting cancer cell proliferation, migration, and invasion, possibly by downregulating miR-19a through methylation. Therefore, overexpression of circ-MYBL2 may serve as a potential target to treat PA.

## Supplementary Material

Supplemental Material
