# Supplementary material for: MM-associated circular RNA downregulates microRNA-19a through methylation to suppress proliferation of pancreatic adenocarcinoma cells
Source: Bioengineered. 2022 Apr 7;13(4):9294–300. doi: 10.1080/21655979.2022.2051815 (PMC9161914; doi:10.1080/21655979.2022.2051815)
Supplement: Supplemental Material [file KBIE_A_2051815_SM6841.zip › supplementary/downloadFromZipFile.pdf]

复旦大学华山医院伦理委员会

科研论文审批意见书

申请人姓名： 徐江慧

申请审批项目名称： circ-MYBL2 在胰腺癌中的作用和机制研究

申请日期： 2018 年 4 月 20 日

批准日期： 2018 年 4 月 20 日

经伦理委员会审查，本项目所提交的研究方案等资料符合医学伦理原则和赫尔辛基宣言的各项要求，研究设计具有科学根据，没有给受试者带来不必要的危险，人类组织提取的途径是规范的，对受试者的安全和隐私给予了最大限度的保护。所选择的动物品种、等级、数量、规格合适。实验中善待动物，给予麻醉和镇痛处理，实验后给予安乐死，死后动物无害化处理，没有对环境带来危害，实验符合动物实验研究伦理标准。同意该项目产生的论文投稿。

复旦大学华山医院研究伦理委员会

复旦大学附属华山医院 伦理审查委员会 HIRB

2018 年 4 月 20 日
