# Supplementary figures and images for: MM-associated circular RNA downregulates microRNA-19a through methylation to suppress proliferation of pancreatic adenocarcinoma cells
Source: Bioengineered. 2022 Apr 7;13(4):9294–300. doi: 10.1080/21655979.2022.2051815 (PMC9161914; doi:10.1080/21655979.2022.2051815)

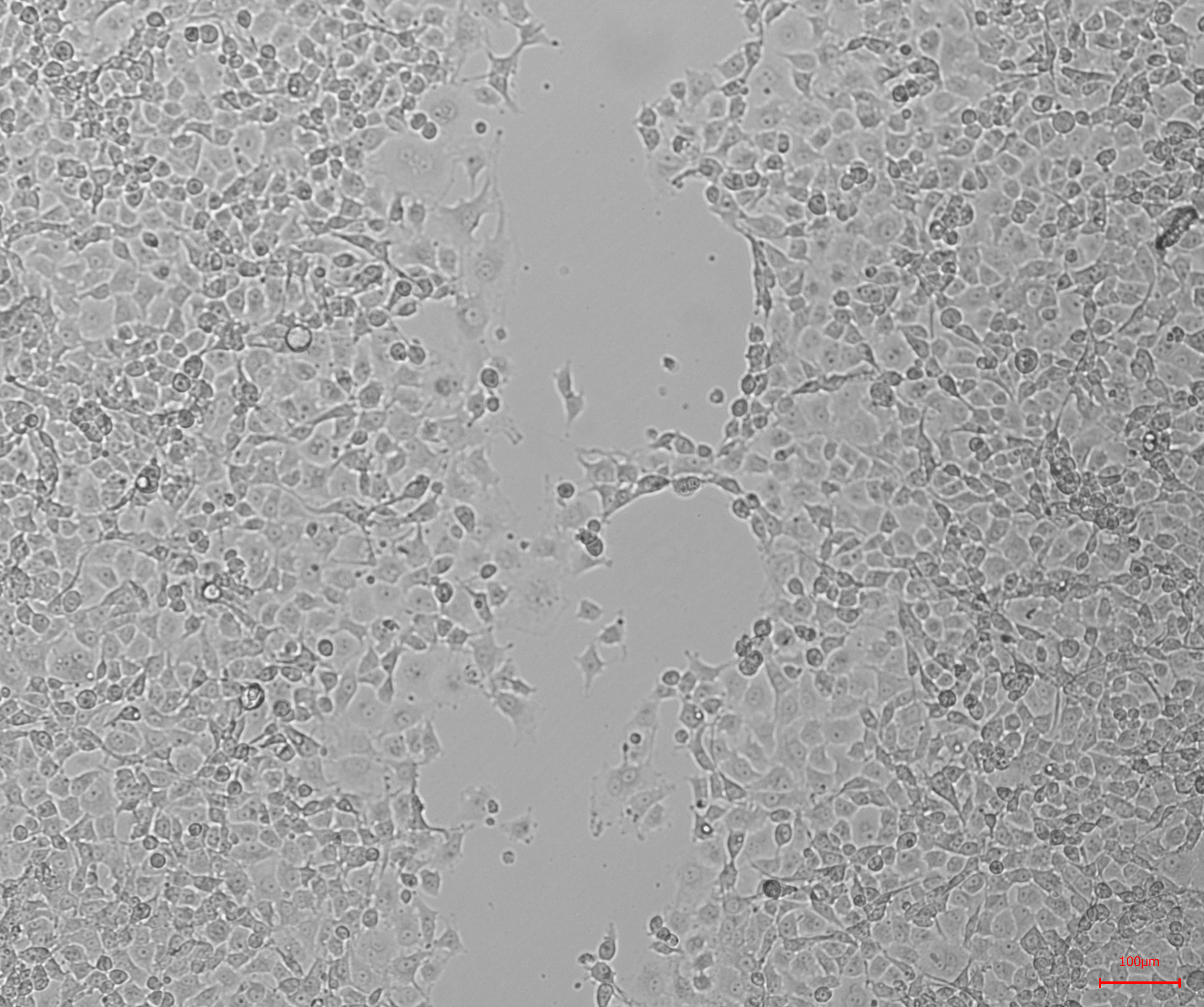

Supplement: Supplemental Material [file KBIE_A_2051815_SM6841.zip › supplementary/circ-MYBL2.tif]

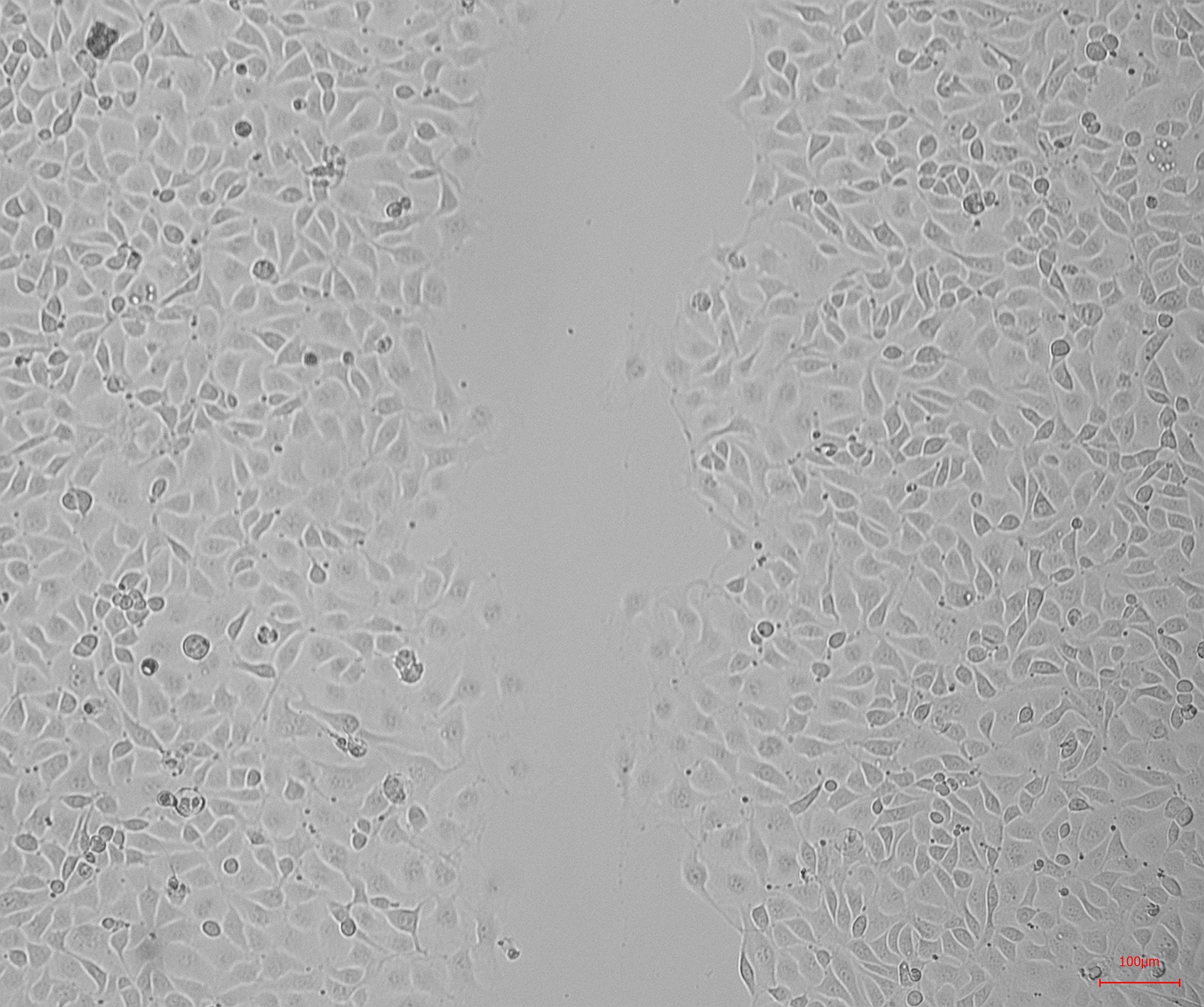

Supplement: Supplemental Material [file KBIE_A_2051815_SM6841.zip › supplementary/circ_MYBL2.jpg]

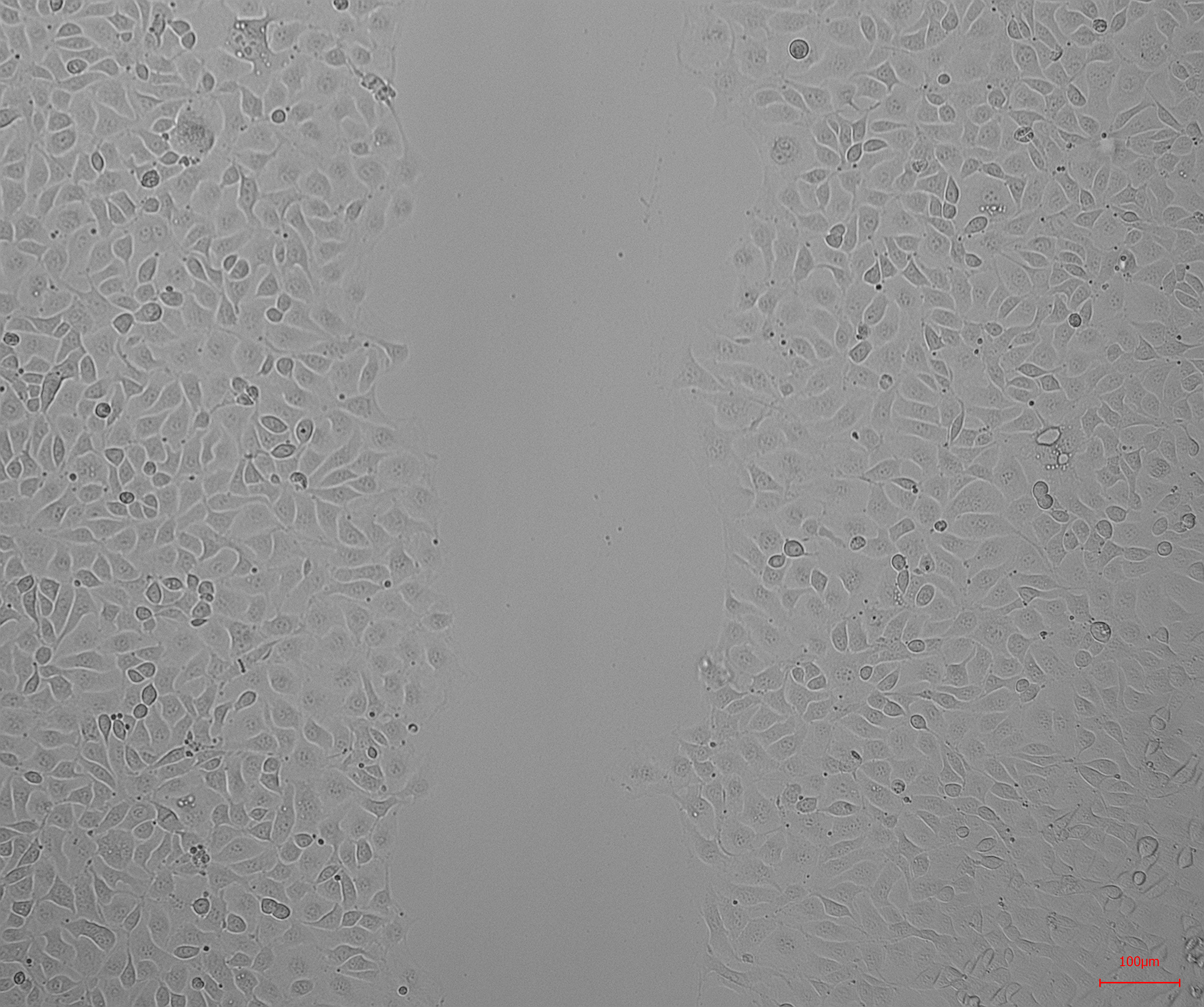

Supplement: Supplemental Material [file KBIE_A_2051815_SM6841.zip › supplementary/Control.tif]

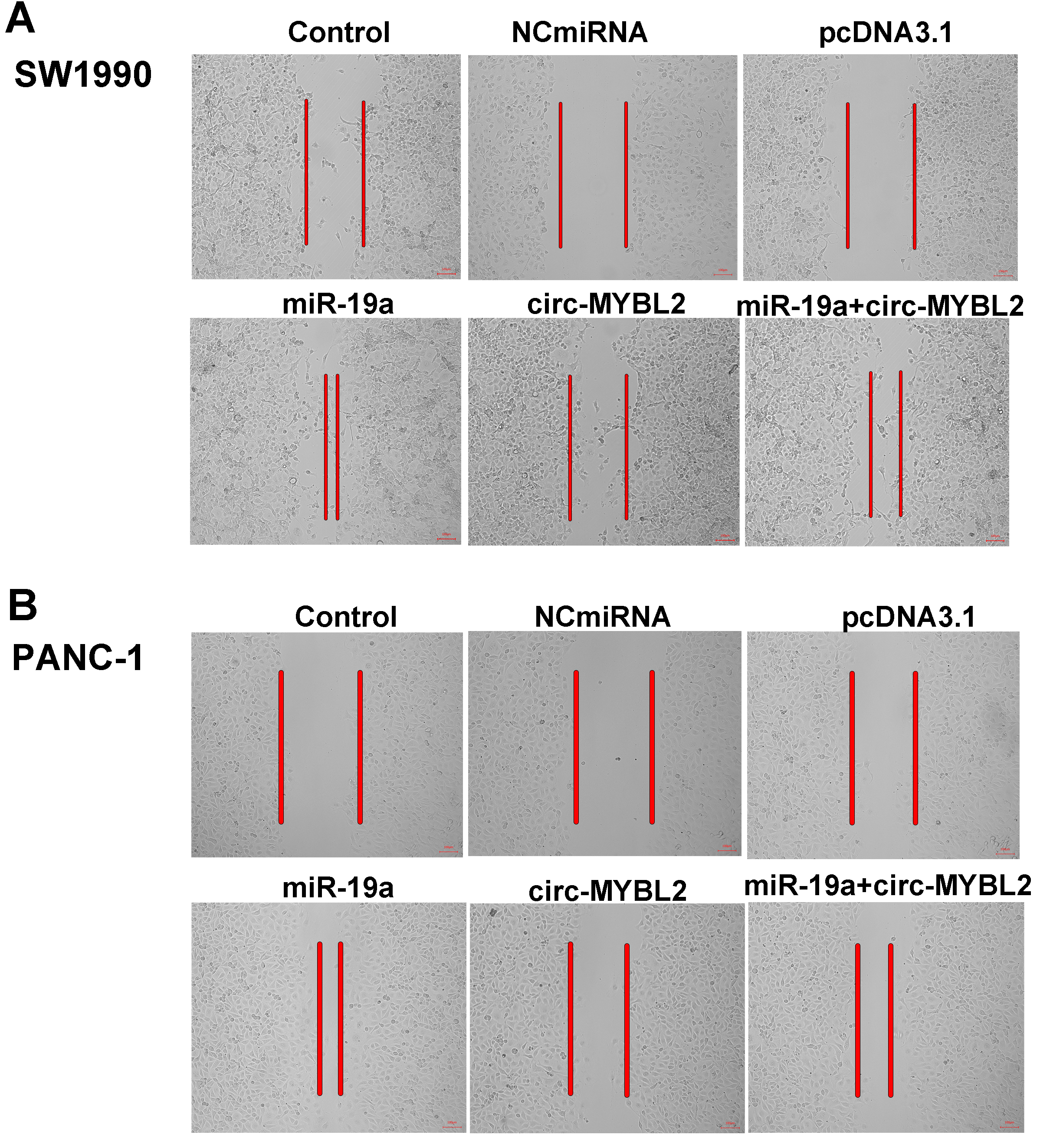

Supplement: Supplemental Material [file KBIE_A_2051815_SM6841.zip › supplementary/Figure S1.tif]

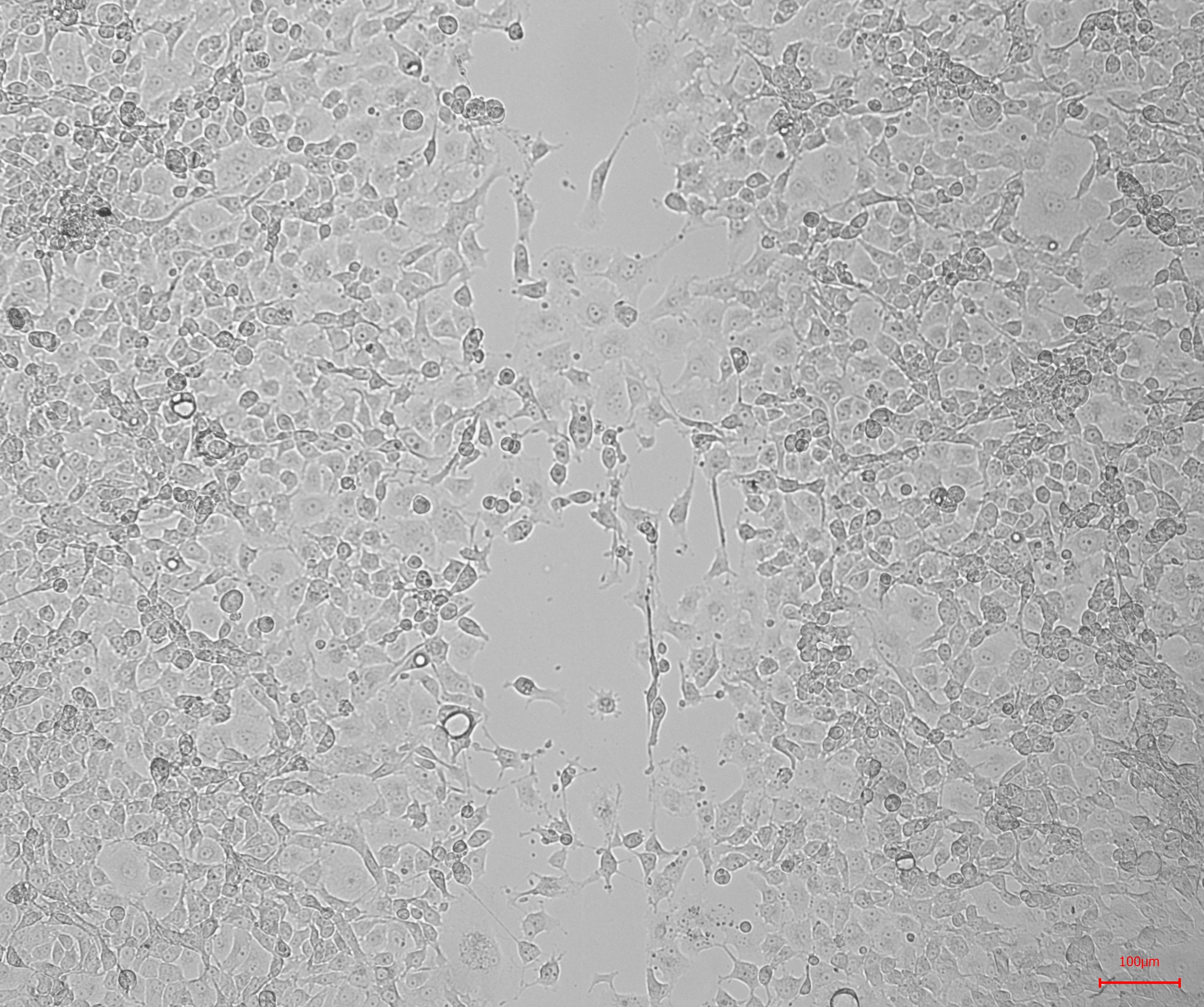

Supplement: Supplemental Material [file KBIE_A_2051815_SM6841.zip › supplementary/miR-19a.jpg]

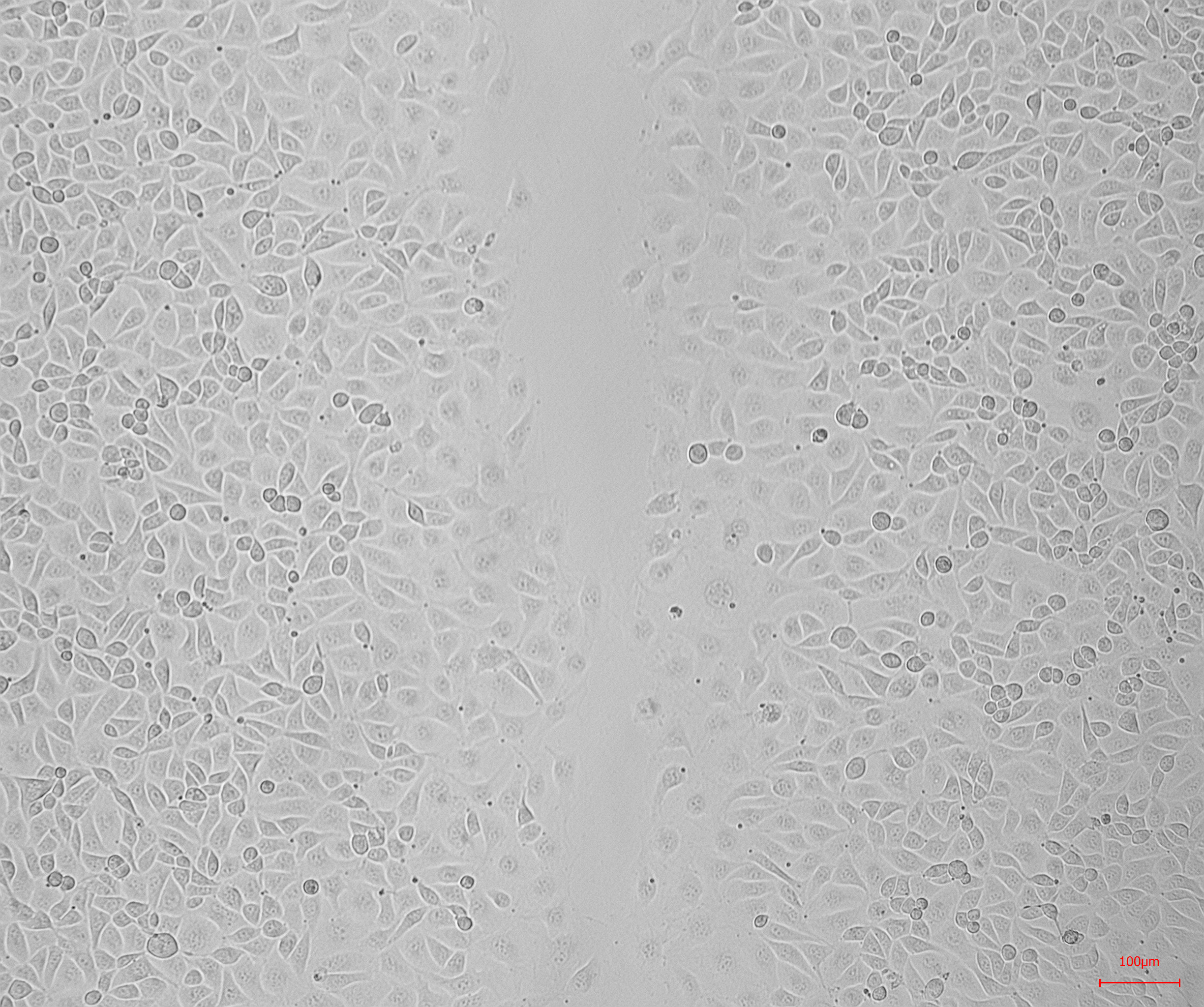

Supplement: Supplemental Material [file KBIE_A_2051815_SM6841.zip › supplementary/miR-19a.tif]

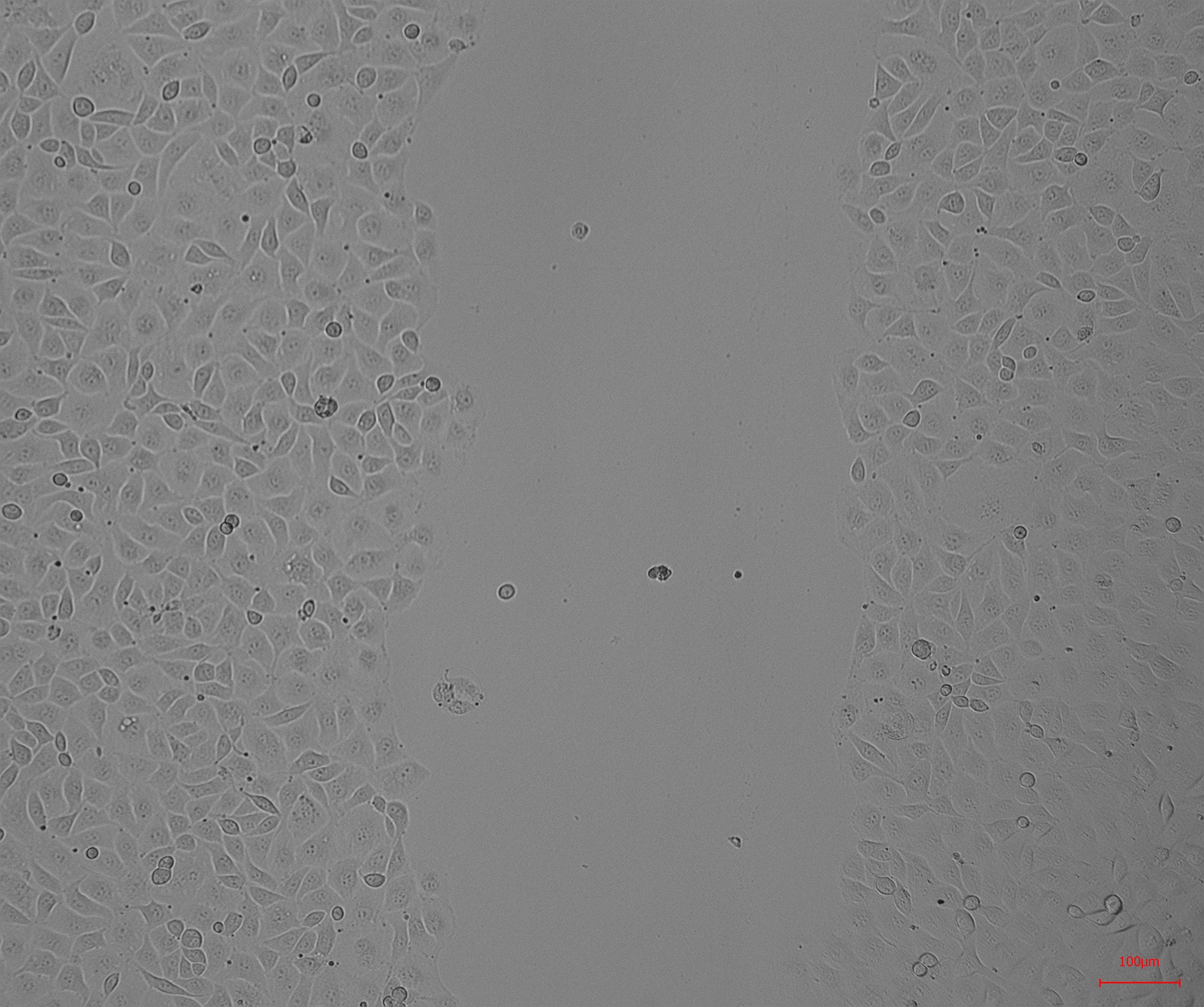

Supplement: Supplemental Material [file KBIE_A_2051815_SM6841.zip › supplementary/NC miRNA.jpg]

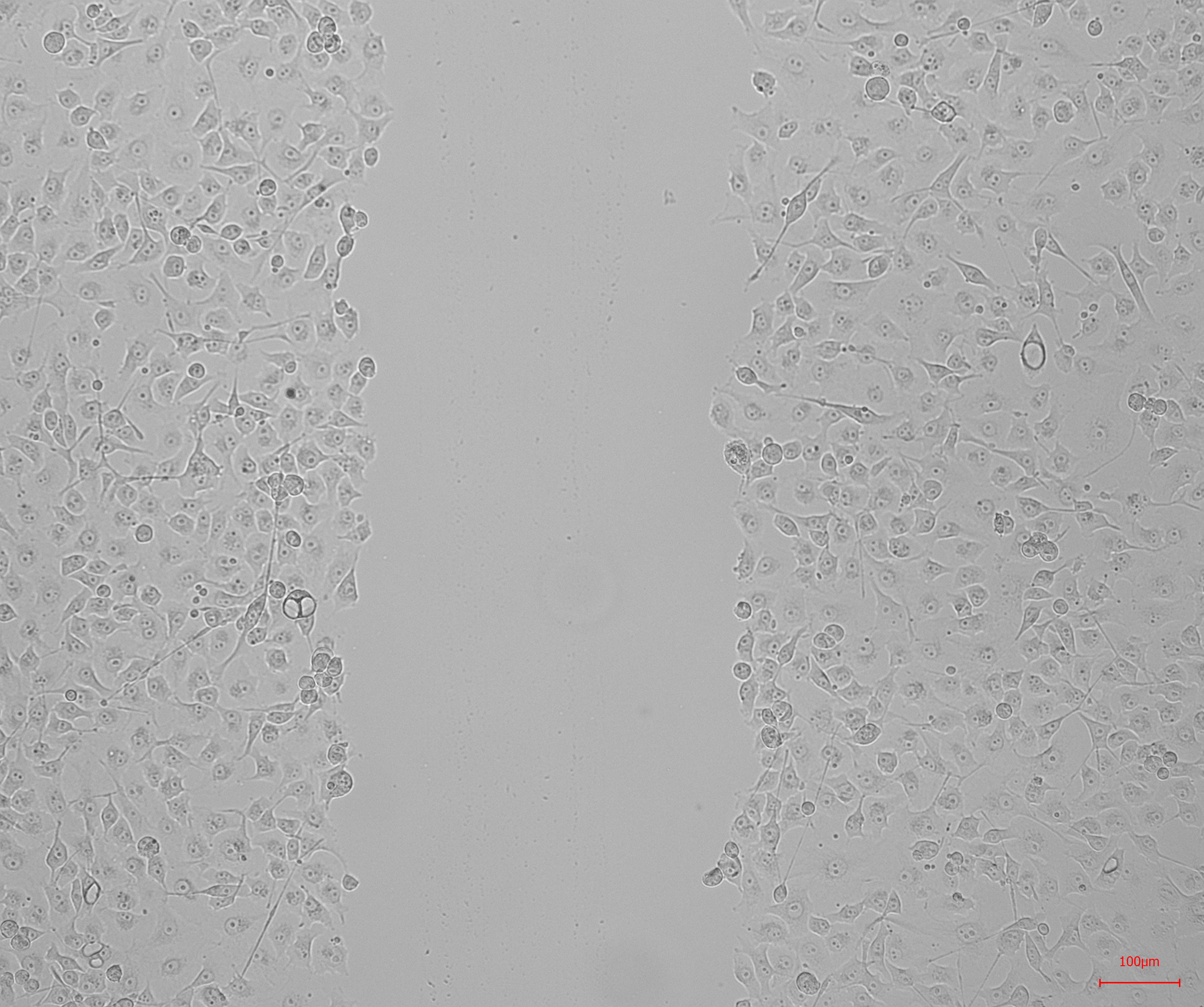

Supplement: Supplemental Material [file KBIE_A_2051815_SM6841.zip › supplementary/NCmiRNA.tif]

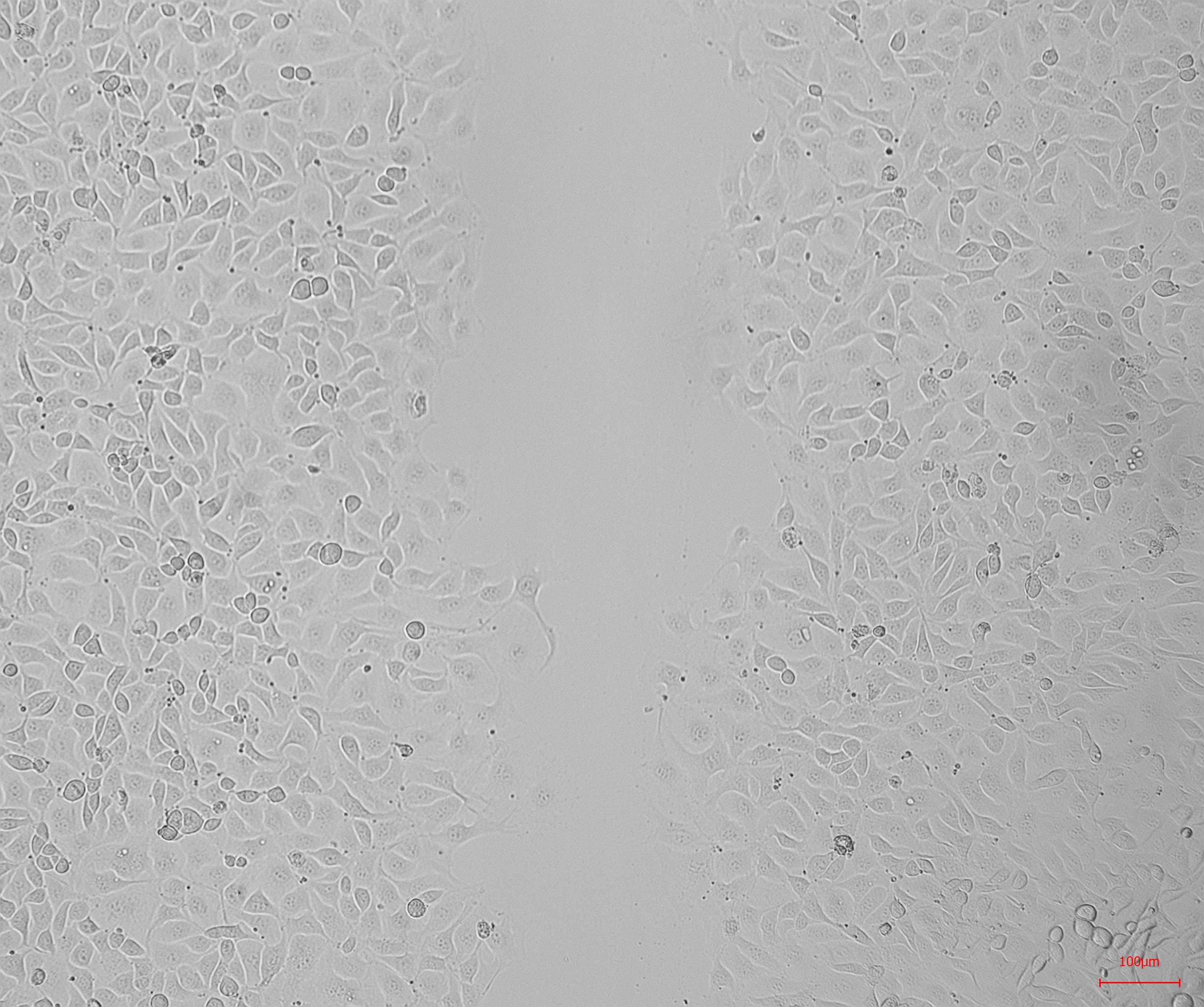

Supplement: Supplemental Material [file KBIE_A_2051815_SM6841.zip › supplementary/pcDNA3.1.jpg]
